# Running on empty – a nationwide large-scale examination of compulsive exercise in eating disorders

**DOI:** 10.1186/s40337-018-0197-z

**Published:** 2018-06-12

**Authors:** Elin Monell, Johanna Levallius, Emma Forsén Mantilla, Andreas Birgegård

**Affiliations:** 1 Centre for Psychiatry Research, Department of Clinical Neuroscience, Karolinska Institute, Stockholm, Sweden; 2 Stockholm Health Care Services, Stockholm County Council, Stockholm, Sweden

**Keywords:** Eating disorder, Compulsive exercise, Outcome, Females, Males

## Abstract

**Background:**

Compulsive exercise (CE) has been the neglected “Cinderella” among eating disorder (ED) symptoms, even though it seems to impact severity, treatment and outcome. This prompted a large-scale and systematic examination of the impact of CE in a representative ED sample.

**Methods:**

CE was examined in over 9000 female and male patients from a clinical ED database (covering out-patient, day and/or residential treatment) with respect to prevalence, ED diagnosis, ED symptoms, clinical features, patient characteristics, and outcome at 1-year follow-up. Relationships between changes in CE behavior and remission were also examined.

**Results:**

CE was a transdiagnostic symptom, present in nearly half of all patients (48%). It was associated with greater overall ED pathology, particularly dietary restraint, and negative perfectionism. Initial CE did not impact remission rate, but patients continuing or starting CE during treatment had considerably lower remission rates compared to patients who never engaged in, or ceased with, CE. Results were comparable for females and males.

**Conclusions:**

At baseline, there were few differences between patients with and without CE, except a somewhat higher symptom load for patients with CE, and CE did not predict ED outcome. However, how CE developed during treatment to 1-year follow-up considerably impacted remission rates. We strongly recommend CE to be systematically assessed, addressed, and continuously evaluated in all ED patients seeking treatment.

## Plain English Summary

Compulsive exercise (CE) and its relations to eating disorder (ED) symptoms, clinical features, and outcome were examined in over 9000 female and male patients from a nation-wide clinical ED database. CE was reported by almost half of all patients, and prevalent in all ED diagnoses. There were few differences between patients with and without CE, except that patients with CE at baseline reported a slightly higher symptom load. CE at baseline did not affect prognosis, but how CE developed considerably impacted prognosis. Patients continuing or starting CE during treatment had much lower remission rates, compared to patients who ceased with (or never had) CE. Therefore, we strongly recommend CE to be systematically assessed, addressed, and continuously evaluated in all patients with EDs seeking treatment.

## Background

The compulsive features of exercise in eating disorders (EDs) have gradually been given increased attention [1]. Therefore, exercise in ED is nowadays generally defined as *compulsive exercise* (CE), encompassing aspects of weight and shape regulation, affect regulation, and compulsivity [2]. CE is a common symptom in various ED presentations, implicated in ED etiology and maintenance [3–5]. CE has been related to more severe ED pathology [6–8], longer in-patient treatment [9], and increased risk for relapse in anorexia nervosa (AN) [10]. Hence, CE seems to imply more serious and long-lasting ED. In adolescents, CE consistently relates to more ED pathology, particularly dietary restraint, regardless of diagnosis, and failure to successfully treat CE may predict worse ED outcome [11]. Research regarding the impact of CE across diagnostic categories and in relation to outcome in adults is scarce. We therefore aimed to extend upon Levallius, Collin and Birgegård’s study on adolescents [11], by examining associations between CE, related clinical features and different EDs, as well as longitudinal associations to outcome, in the largest adult patient sample to date.

CE prevalence in various ED samples at different treatment levels ranges between 39 and 46% [6, 8, 12]. Prevalence findings in different ED subgroups are disparate. In the largest studies, the highest CE prevalence was found among purging AN patients (AN-P) [8] and restricting AN patients (AN-R) [6]. Conversely, among adolescent patients, CE was most common in bulimia nervosa and ED not otherwise specified (BN and EDNOS) as opposed to AN [11]. In smaller samples, one study found CE to be more frequent in AN compared to BN [3], while another found more CE in AN-P and BN compared to AN-R [13]. Some studies have found no diagnostic differences [14, 15]. Regarding CE attitudes (i.e. compulsive and negative attitudes toward exercise), patients with BN and EDNOS rated higher problematic attitudes compared to AN in one study [16], whereas no such differences were found in another [17]. The vast majority of studies mainly included female patients, while knowledge on CE in males with an ED is sparse. A large study on ED patients, drawn from the same database as the present study, indicates slightly lower CE rates among men compared to women (40% vs. 45%) [12]. In adolescent ED, boys also displayed somewhat lower CE rates than girls (38% vs. 44%) [11].

Regardless of diagnosis and level of care, CE behavior and attitudes seem consistently related to greater ED psychopathology [6–8, 11, 18]. CE seems particularly associated with more dietary restraint [6, 7, 11, 19, 20]. Further, some studies indicate that adult patients with CE tend to report less binging and purging behaviors compared to patients without CE [6, 14, 20], while no such associations were found among adolescents [11]. However, since sample composition differs between studies, and some comparisons were underpowered, replications in larger samples are needed.

There is no conclusive evidence on associations between CE and patient characteristics such as age, ED onset, ED duration, and body mass index (BMI). Age seems mainly unrelated to CE [6, 7, 13], though CE patients were slightly younger than non-CE patients in one AN-dominated sample [8]. Two studies indicate that some elements of CE might be associated with earlier ED symptom onset among patients with AN [14, 18], while no such association was found in adolescent patients [11]. Where reported, CE and ED duration seem unrelated [6, 8, 9]. Regarding CE and BMI, studies have yielded divergent results. Patients with CE had higher BMI in one study [15], lower BMI in another [20], and three other studies found no association between CE and BMI [6, 7, 13]. These inconsistencies may reflect differences in sample composition.

Associations between CE and psychiatric comorbidity and problematic clinical features are similarly inconclusive. Presence of CE has been related to more negative perfectionism [8, 11], increased suicidality [11, 21], and more self-injury [20]. Presence of CE has further been related to increased obsessive/compulsive symptoms and/or traits [8, 18], but the opposite result has also been found [19]. Some research found that presence of CE was related to increased anxiety [8, 15] and depression [15], while no such associations were found by others [6, 11, 16]. Lastly, while ED is usually related to lower self-esteem, results from two studies in AN-samples found that presence of CE indicated higher self-esteem [19, 22], although the opposite has also been reported [11].

A few studies on ED inpatients have examined CE in relation to treatment and outcome variables. In AN-samples, CE was related to increased risk for treatment dropout [23], increased risk for relapse [10], and shorter time to relapse [24]. It has also been related to longer in-patient treatment in a mixed sample (AN, BN, EDNOS) [9]. In another mixed inpatient sample (AN, BN, EDNOS), presence of CE at discharge, but not admission, was related to worse outcome [6]. Relatedly, in a similar sample, reduction in CE attitudes and behaviors during inpatient treatment has been related to improvements in ED psychopathology, for patients classified as compulsive exercisers [20]. In the two latter studies, CE was unrelated to treatment dropout. In Levallius’ et al. large adolescent ED sample (AN, BN, and EDNOS) [11], where patients could receive both in- and/or outpatient care, presence of CE at follow-up, but not at admission, was likewise related to worse outcome. Knowledge of CE and outcome in adult patients across levels of care is lacking.

### Aims and hypotheses

To address these gaps, and to extend the study by Levallius et al. focusing on adolescents [11], we aimed to examine relations between self-reported CE and ED diagnosis, key ED symptoms, related clinical features, and 1-year outcome in adult patients with ED receiving treatment as usual (TAU) in out-patient, day and/or in-patient treatment settings. We expected CE prevalence in line with previous research (i.e. 36–45%). We further predicted associations between presence of CE and increased overall ED symptom level and increased dietary restraint, as well as increased suicidality, negative perfectionism and compulsivity. Lastly, we hypothesized that CE at admission would not impact remission rate, but that CE at follow-up (i.e. patients not ceasing CE, or starting CE during treatment) would be associated with a lower remission rate. No expectations or hypotheses were formulated regarding CE prevalence for each diagnosis (due to inconsistent findings), gender differences, relations between presence of CE and levels of binge eating and purging, associations between CE and age, ED onset, ED duration, or BMI, nor associations with depression, anxiety and self-esteem.

## Methods

### Participants and procedure

Data came from the Stepwise database, a clinical database for specialized ED treatment units in Sweden [25], representing the full range of treatment modalities (e.g., medical, psychological, pedagogical, nutritional, social, physical), length, and intensity. Out-patient treatment alone is most common for adult patients (approx. 60% of patients); day treatment and residential care are given to about one fourth and one fifth of patients, respectively [26]. Stepwise includes data from patients aged 7–81, with the full range of DSM-IV ED diagnoses [27] entering treatment since 2005. Stepwise inclusion criteria are medical or self-referral to a treatment unit, a DSM-IV ED diagnosis, and intention to treat from the clinic. Stepwise assessment, including interviews and self-rated questionnaires, is performed by trained ED professionals within the patients 3rd visit to the treatment unit (for inpatients, during the 1st week). The whole assessment takes around 45 min. *Age at onset* was defined as patients’ retrospective report on symptom debut, and *ED duration* was computed as the difference between that definition and patient age at assessment. Age at onset was only available for patients entering treatment since 2013 (*N* = 4382). Patients with and without such information did not differ significantly (see Statistical analysis) in self-rated ED psychopathology, age, BMI and diagnostic distribution. *ED remission* was defined as not meeting diagnostic criteria for an ED diagnosis at 1-year follow-up assessment. Only a subset of patients had follow-up data (women: *N* = 3073; men: *N* = 114), where missing data indicate treatment drop-out (less common) or dropout from follow-up (more common). Patients with or without follow-up assessment did not differ significantly on the variables stated above (data not shown).

All patients registered since the start of the database in March 2005 until October 2017 were extracted (*N* = 17,462). The following exclusions were then performed: empty registrations (14 cases), age < 18 (5599), no research consent (400), incomplete registrations (118), no self-ratings (313), no diagnostic data (19), no ED diagnosis after assessment (571), double/multiple registrations (796; last registration kept), and lastly, cases with types of ED not otherwise specified (EDNOS) not relevant for this study (517: “EDNOS-chewing and spitting”=78; “EDNOS-other” = 439). Nine thousand one hundred seventeen patients remained in the final sample. During the study time-span, an increasing number of all specialized ED units registered in the Stepwise database, with approximately 90% of all such units in Sweden reporting stably from 2012, and the coverage ratio within these units was high (> 80%). Ethical approval was granted by the regional review board (No. 2009/1298-31/1, EPN Stockholm).

### Measures

*Structured Eating Disorder Interview* (SEDI) [28] is a semi-structured interview used to support clinical ED diagnosis of the patient according to the criteria in DSM-IV. The SEDI was specifically developed for the Stepwise system and has a high concordance to the Eating Disorder Examination (EDE) interview; 90.3% concerning the presence of ED (sensitivity = .91, specificity = .80), and 81.0% concerning specific ED subdiagnosis (Kendall’s Tau-b .69, *p* < .001) [28].

*Eating Disorder Examination questionnaire* (EDE-Q) [29] consists of 36 items measuring key ED symptoms the last 28 days. Items are rated on a 7-degree scale and provide a Global Score and the subscales *Restraint*, *Eating Concerns*, *Weight Concerns* and *Shape concerns*. Higher scale scores indicate greater symptom severity. It also provides information on presence and frequency of behavioral symptoms. This study used the Global Score to indicate overall ED pathology and severity, *Restraint* to indicate degree of dietary restraint in order to influence shape or weight, and information on *binge eating*, *purging*, and *exercise*. Many previous studies have based CE on frequencies of driven/excessive exercise as defined by EDE and EDE-Q while recently, others have used measures capturing the psychological aspects of CE. However, strong correlations between EDE-Q driven exercise frequency item and established CE measurements have been reported [18]. This study uses the presence/absence of driven exercise (*“Have you exercised hard to control weight and shape?”*) as a proxy for CE, which has previously been done in an adolescent ED sample [11]. Driven exercise frequency is used additionally for some specific analyses. The EDE-Q has good psychometric properties [30], and the Swedish version of the EDE-Q has satisfactory validity and reliability [12]. The EDE-Q is a mandatory self-report in the Stepwise system.

*Structural Analysis of Social Behavior – introject questionnaire* (SASB; Benjamin [31]) consists of 36 items measuring self-directed behaviors. Items are rated on a 0–100 scale (10-point increments), forming eight clusters (comparable to subscales) in a circumplex organized on two axes: Affiliation ranging from self-love to self-attack, and Autonomy ranging from spontaneity/letting go to self-control. As previously done by Levallius et al. [11], this study used two variables from the SASB: the Affiliation vector (computed as a weighted summary score of items belonging to the Affiliation axis; range − 100–100) as a proxy for *self-esteem* [32]; and the cluster Self-blame (including self-criticism and accusation, self-blame for mistakes, and negative interpersonal comparisons; range 0–100) as a proxy for *negative perfectionism*. Higher scores indicate better self-esteem, and more negative perfectionism respectively. The SASB Introject has good psychometric properties, and the Swedish translation in the Stepwise system has acceptable reliability (mean α = .75) [33]. The SASB introject is mandatory in the Stepwise system.

*Comprehensive Psychopathological Rating Scale – self-rated version of the affective scales* (CPRS-S-A) [34] consists of 19 items measuring self-rated psychiatric symptoms over the past 3 days. Items are rated on a 0–3 scale (0.5 point increments). Items form three subscales, all used in this study, for *anxiety* (9 items), *depression* (9 items), and *compulsivity* (8 items), where some items overlap and belong to more than one scale. One single item inquires the patient’s will to live and perception of suicide as a viable option, used in this study as a measure of *suicidality*. Higher scores indicate greater symptom severity. The CPRS-S-A has good psychometric properties [34]. CPRS-S-A is mandatory in the Stepwise system.

*Structured Clinical Interview for DSM-IV-Axis I Disorders* (SCID-I) [35] is a semi-structured interview used to examine possible clinical comorbid psychiatric Axis I diagnoses of the patient according to the criteria in DSM-IV [27]. For this study, information on presence/absence of the following disorders was used for additional analyses: *ongoing depressive episode*, *generalized anxiety disorder* (GAD), and *obsessive/compulsive disorder* (OCD). SCID-I is part of the Stepwise system and is administered to all patients > 18 years.

### Statistical analysis

Analyses were conducted using SPSS Version 25 for Mac. All analyses were performed separately for women and men. Chi-square (*χ*^2^) was used to examine general and diagnosis-specific CE-prevalence. Analyses of variance (ANOVAs) or *χ*^2^ were used for comparisons between CE and non-CE patients on ED symptoms (EDE-Q Global, Restraint, binge and purge frequency), patient characteristics (age, age at onset, ED-duration, and BMI), and clinical features/disorders (anxiety, depression, compulsivity, suicidality, self-esteem, and negative perfectionism). Additionally, to test whether CE frequency had any relation to the variables of interest, Spearman correlations were conducted (since data were non-normally distributed). Due to high intercorrelations for some variables, this was followed by a multiple linear regression analysis with all variables as independent and CE frequency as dependent variable. To adjust for outliers regarding ED behavior frequency, unreasonably high frequencies (e.g. more than 100 exercise sessions in 1 month) were adjusted by inspecting the frequency distribution for discontinuities, and adjusting the extreme values to one above the next highest, consecutively (e.g. 120 became 101, 160 became 102 etc.). This method avoided large gaps in the distribution due to single extreme values (similar method in Ekeroth, Clinton, Norring & Birgegård [36]; but patients were retained rather than deleted). Analyses including behavior frequencies were performed solely within patients reporting such behavior (i.e. patients not reporting it were excluded).

Relations between ED remission and CE prevalence and frequency at admission respectively were examined by *χ*^2^ and logistic regression. To examine the effect of change in CE behavior on ED remission, combinations of CE prevalence at admission and at follow-up formed four patient categories: *never* (no CE either at admission or follow-up), *ceases* (CE at admission but not follow-up), *starts* (no CE at admission but at follow-up), and *continues* (CE at admission and follow-up both), which were related to ED remission by *χ*^2^. Additionally, the effect of change in CE behavior on ED severity (i.e. EDE-Q Global Score) at admission and follow-up were examined by ANOVAs.

Effect sizes were computed: phi (Φ) for *χ*^2^, and *η*^*2*^_*partial*_ for ANOVAs, and were considered small Φ > .10/ *η*^*2*^_*partial*_ > .01, medium Φ > .30/ *η*^*2*^_*partial*_ > .06, and large Φ > .50/ *η*^*2*^_*partial*_ > .14. Correlations and *β* coefficients were considered small >.10. Due to multiple comparisons and tests, significance level was set to *p* < .001 for analyses among women. For analyses among men, the significance level was *p* < .05 due to the considerably smaller sample size. Only significant pairwise comparisons with ≥ small effect sizes will be reported.

In Levallius’s et al. adolescent sample [11], there was an effect of denial of illness on several associations, such that there were smaller differences between patients with or without CE when patients exhibiting denial of illness were excluded (these patients were less likely to report both CE and other symptoms, and may therefore skew analyses). Therefore, all analyses that produced significant and meaningful effects were also conducted without cases possibly exhibiting denial of illness. The definition of denial of illness is patients scoring below diagnosis-, age-, and gender-adjusted clinical cut-offs on the EDE-Q Global Score, using Clinical Significance (CS) calculations as described in Ekeroth and Birgegård [37].

## Results

### Sample characteristics

Of the total sample of 9117 patients, 96.3% were female, and age ranged between 18 and 81 (*M* = 26.3, *SD* = 8.2). CE was present in 48.2% of the female patients at admission, and in 45.5% of the male patients. CE prevalence differed significantly between diagnoses (AN, BN, BED, EDNOS) among both female (*χ*^2^(3, 8740) = 428.86, *p* < .001, Φ = .222), and male patients (*χ*^2^(3, 334) = 15.93, *p* = .001, Φ = .218), where CE was most frequent in patients with EDNOS followed by BN (see Table 1). Within the female AN-group, additional analyses showed that 38.8% reported CE among those classified as AN-R (*N* = 1078), compared to 43.7% among those classified as AN-BP (*N* = 490). Within the male AN-group, CE was reported by 30.4% of the AN-R (*N* = 46) and 53.3% of the AN-BP patients (*N* = 15).

**Table 1:** Diagnostic distribution, presence of CE, CE frequency *M (SD)*, presence of OBE and purging per diagnosis for women and men separately. CE frequency only for patients reporting such behavior (women: *N* = 4223; men: *N* = 152). Remission rates at 1-year follow-up (women: *N* = 3060; men: *N* = 113)

		AN	BN	BED	EDNOS	Total
*N*	Women	1522 (17.4%)	2849 (32.6%)	628 (7.2%)	3741 (42.8%)	8740
	Men	61 (18.3%)	91 (27.2%)	36 (10.8%)	146 (43.7%)	334
CE (%)	Women	40.3	51.1	13.1	55.1	48.2
	Men	36.1	45.1	22.2	55.5	45.5
CE frequency	Women	18.8 (16.0)	14.8 (9.7)	9.5 (6.6)	16.3 (10.5)	16.0 (11.3)
	Men	19.5 (16.9)	16.9 (10.8)	10.3 (5.8)	16.9 (11.6)	16.9 (12.1)
OBE (%)	Women	32.5	75.3	78.0	45.9	55.5
	Men	27.9	77.2	75.0	45.2	54.0
Purging (%)	Women	29.7	73.6	8.0	44.3	48.7
	Men	23.0	63.7	33.6	0.0	36.2
Remission rates (%)	Women	40.5	56.5	70.9	57.9	55.2
	Men	66.7	57.6	66.7	61.0	61.9

*CE* compulsive exercise, *OBE* objective binge-eating episode, *AN* anorexia nervosa, *BN* bulimia nervosa, *BED* binge eating disorder, *EDNOS* eating disorder not otherwise specified. CE frequency reported for the last 28 days

### CE and relations to ED symptoms, patient characteristics and clinical features

Both female and male CE patients had more general ED pathology and more restraint than non-CE patients (small-medium effects; see Table 2). Among female patients with purging episodes, those who also had CE reported fewer purging episodes than those without CE (small effect). When examining mean frequencies among male patients with purging, those with CE also reported fewer purging episodes than those without CE, but this difference did not reach significance. There were no differences in binge frequency in relation to CE presence in either female or male patients.^1^

**Table 2:** Means, standard deviations *M(SD)* and comparisons between CE and non-CE patients on ED symptoms, patient characteristics and clinical features, in women and men separately. Effect sizes reported for significant results, meaningful effects (≥ small) in bold. *N* varies for each analysis due to missing data in one or more variables

		Women					Men				
		Analysis *N*	CE	Non-CE	F	*η* ^2^_partial_	Analysis *N*	CE	Non-CE	F	*η* ^2^_partial_
EDE-Q	Global	8740	4.2 (1.0)	3.6 (1.3)	522.73^***^	**.056**	334	3.6 (1.1)	3.0 (1.3)	18.50^***^	**.053**
	Restraint	8740	4.0 (1.3)	3.1 (1.7)	771.73^***^	**.081**	334	3.4 (1.5)	2.6 (1.7)	19.90^***^	**.057**
	Binge frequency	4870	11.0 (10.7)	12.8 (12.1)	32.42^***^	.007	181	11.8 (11.9)	12.6 (14.4)	.18	
	Purge frequency	4257	16.2 (17.8)	20.8 (23.8)	51.53^***^	**.012**	121	19.5 (21.5)	26.3 (33.7)	1.72	
Patient characteristics	Age	8740	25.3 (7.1)	27.1 (8.9)	109.16^***^	**.012**	334	27.9 (9.6)	27.7 (10.1)	.13	
	Age at onset	4193	16.2 (4.5)	16.1 (5.3)	.06		178	20.1 (8.0)	17.4 (5.6)	7.18^**^	**.039**
	Duration	4193	9.2 (7.8)	11.3 (9.4)	57.57^***^	**.014**	178	8.0 (8.0)	10.1 (10.7)	2.16	
	BMI	8736	21.4 (4.2)	22.9 (7.2)	141.22^***^	**.016**	334	23.4 (6.3)	24.5 (9.0)	1.64	
CPRS	Depressiveness	8740	11.1 (4.7)	10.6 (4.8)	23.60^***^	.003	334	10.4 (4.9)	9.9 (4.8)	.88	
	Anxiety	8740	10.0 (4.3)	9.5 (4.3)	21.96^***^	.003	334	8.9 (4.4)	8.7 (4.2)	.15	
	Compulsivity	8740	9.6 (4.3)	9.0 (4.3)	52.92^***^	.006	334	9.2 (4.6)	8.4 (4.5)	2.79	
	Suicidality	8735	1.7 (1.4)	1.6 (1.5)	2.47		332	1.6 (1.4)	1.6 (1.5)	<.01	
SASB	Self-esteem	8740	−15.6 (34.5)	−12.2 (36.4)	19.66^***^	.002	334	−5.0 (39.3)	−2.0 (39.1)	.51	
	Negative perfectionism	8740	60.0 (22.5)	55.2 (23.7)	94.97^***^	**.011**	334	54.3 (24.4)	48.3 (24.4)	5.00^*^	**.015**

^*^ = *p* < .05; ^**^ = *p* < .01; ^***^ = *p* < .001. *CE* compulsive exercise, *ED* eating disorder, *EDE-Q* Eating Disorder Examination Questionnaire, *CPRS* Comprehensive Psychopathological Rating Scale, *SASB* Structural Analysis of Social Behavior. Binge and purge frequency reported for the last 28 days

Female CE patients were slightly younger, had a slightly shorter ED duration, and had a slightly lower BMI than non-CE patients (see Table 2). There were no differences in any of these variables for male patients, but male CE patients were on average 3 years older at ED onset compared to non-CE patients (small effect). There was no difference in ED onset in female patients. There were no differences between CE and non-CE patients on any clinical feature, except negative perfectionism (see Table 2).^2^ Here, both female and male CE patients had more negative perfectionism than non-CE patients (small effects).

CE frequency was weakly correlated to almost all variables examined above (Spearman rho’s = .101–.286; results not shown), except age, age at onset, and duration (no correlations). When examined by multiple regression analysis, only EDE-Q restraint level was independently related to CE frequency (*β* = .202, *t* = 6.15 *p* < .001; *N* = 2142), such that more restraint indicated a higher CE frequency.

### CE, changes in CE behavior, and relations to outcome

Overall, there was a significant difference in remission rates between diagnoses for female patients, with the lowest in AN and highest in BED, respectively (*χ*^2^(3, 3060) = 80.06, *p* < .001, Φ = .162). General remission rate for male patients was slightly higher compared to females with similar remission rates across all diagnoses (*χ*^2^(3, 113) = .65, *p* = .884). Among female patients, remission rate did not significantly differ between patients with or without CE at admission (53.2% vs. 57.2%; *χ*^2^ (1, 3057) = 4.87, *p* = .027). Nor did CE-frequency at admission relate to remission to any great extent; one additional CE episode/month increased the odds of remaining ill by 2.5% (*p* < .001, Cox & Snell *R*^2^ = 1.7%, *N* = 1492). Male patients with CE at admission seemed to have a somewhat lower remission rate than non-CE patients (54.9% vs. 67.7%; *χ*^2^ (1, 113) = 1.96, *p* = .162), and initial CE frequency seemed to increase the odds of remaining ill by 3.8% (*p* = .107, Cox & Snell *R*^2^ = 6.1%; *N* = 51). As there were few male patients with follow-up data, analyses need to be considered with caution.

Among female patients, there were distinct effects of changes in CE behavior from admission to follow-up on remission rates (*χ*^2^(3, 2862) = 209.94, *p* < .001, Φ = .271). Patients who never engaged in, or ceased with, CE remitted roughly twice as often as those who started or continued with CE during treatment (see Table 3). There were differences in initial overall ED symptomatology depending on CE change category (*F*(3, 2871) = 66.51, *p* < .001, *η*^*2*^_*partial*_ = .065), where patients who continued CE displayed highest initial symptom level. There were also pronounced differences in follow-up ED symptomatology (*F*(3, 2871) = 156.29, *p* < .001, *η*^*2*^_*partial*_ = .140), where patients who never engaged in, or ceased with, CE had considerably lower follow-up symptom level than patients who started or continued with CE. Among male patients, there was no significant effect of CE change category on remission rate (*p* = .730), and descriptively, only patients who continued with CE seemed somewhat worse off than others.

**Table 3:** Remission rates and symptom ratings by change in CE behavior from admission to 1-year follow-up for women (*N* = 2862) and men (*N* = 101)

CE change category	Women				Men			
	Patient distribution	EDE-Q baseline	EDE-Q follow-up	Remission rate	Patient distribution	EDE-Q baseline	EDE-Q follow-up	Remission rate
Never	44.4%	3.6 (1.2)	2.0 (1.5)	62.1%	51.5%	3.1 (1.1)	1.7 (1.4)	65.4%
Ceases	30.2%	4.1 (1.0)	1.9 (1.4)	64.5%	26.7%	3.7 (1.2)	1.4 (1.3)	63.0%
Starts	6.6%	3.9 (1.1)	3.3 (1.4)	28.2%	5.9%	3.3 (1.4)	2.5 (1.4)	66.7%
Continues	18.8%	4.2 (1.0)	3.3 (1.4)	33.8%	15.8%	3.7 (1.0)	2.3 (1.4)	50.0%

*CE* compulsive exercise, *EDE-Q* Eating Disorder Examination Questionnaire

### Effects of symptom denial

Among female patients, 17.8% displayed possible denial of symptoms, and those were more often found in the non-CE group than in the CE group (22.7% vs. 12.6, suggesting denial of CE as well). Among male patients, there were 18.6% possible deniers, again more often found in the non-CE group (25.3% vs. 10.5%). Exclusion of denying patients increased female CE prevalence to 52.2% and male CE prevalence to 50.0%. It further changed some of the meaningfully significant results reported above. Effect sizes decreased for CE associations to general ED psychopathology (females: *η*^2^_partial_ = .021; males: *p* = .077, *η*^2^_partial_ = .012) and restraint (females: *η*^2^_partial_ = .043; males: *η*^2^_partial_ = .022), and associations to negative perfectionism were no longer meaningful (females: *p* = .001, *η*^2^_partial_ = .002; males: *p* = .439, *η*^2^_partial_ = .002). The effects and overall distribution of remission rates depending on CE change category remained almost identical in female patients (*N* = 2415) and male patients (*N* = 85).

## Discussion

This is the largest study to date examining self-reported CE in adult patients with EDs. The study predominantly replicated the findings of a similar large-scale study on adolescents [11]. CE was reported by almost half of all patients, and prevalent in all diagnoses. The main finding was that patients who continued or started with CE during follow-up had considerably lower remission rates compared to patients who never used or ceased with CE. CE therefore seems a prevalent symptom related to worse ED outcome, warranting due attention in assessment, treatment, and research.

CE was pervasive among ED adults: almost half of all patients reported CE, exceeding hypothesis and previous findings [6, 8, 11, 12]. CE was prevalent in all ED diagnoses with highest prevalence in EDNOS and BN, replicating findings in ED adolescents [11], although CE prevalence in AN may be underestimated due to self-report methodology [23]. Within the AN-group, there was a slightly higher CE prevalence in AN-BP compared to AN-R, as seen in previous studies [8, 13]. CE prevalence in males was slightly lower than in females, as also seen in adolescents [11]. Of note, a 2011 study using the current database found lower overall CE rates but higher binge and purge rates than we did [12], potentially suggesting gradual symptom-shifting among ED patients during this time frame. This would be relevant to investigate further.

As hypothesized, self-reported CE at baseline was associated with greater overall ED pathology and restraint in both genders, in line with previous findings [6–8, 11, 19]. Higher CE frequency indicated increased restraint. Restraint is perhaps most typically associated with AN, but our results indicate higher incidence of CE in EDNOS and BN, thus possibly suggesting dietary restraint to be more common in these groups when CE is present. Female CE patients had slightly lower age, ED duration and BMI. Unexpectedly, male CE patients were on average 3 years older at ED onset than non-CE males. As hypothesized, CE was associated to more negative perfectionism in both genders. Contrary to hypotheses, we found no associations to compulsivity, suicidality or self-esteem, nor any associations to depression or anxiety. Comparisons to previous findings are challenging as sample characteristics, methodology, and CE definition/measurement differs greatly between studies. In the AN-dominated sample by Shroff and colleagues [8], CE patients were also somewhat younger than non-CE patients and CE was associated to negative perfectionism, but unlike our results, CE was also associated to compulsivity and anxiety. When comparing our results with adolescents, findings are mainly corroborated [11]. In sum, our results indicated few meaningful differences between patients with CE and patients without.

It was somewhat puzzling not to find a relation between CE and negative affect, as the anxiolytic effects of exercise in both healthy and clinical populations have been well documented [38]. There is also support for CE functioning as a means of regulating negative affect in ED patients [5, 39, 40]. As such one might expect CE patients to exhibit lower levels of depression and anxiety compared to non-CE patients. Either this compulsive form of exercise does not serve to mitigate distress, or alternately, CE patients experience more distress than non-CE patients to begin with. If the latter is true, CE might actually reduce negative affect in these patients, which also decreases the differences between them and non-CE patients. Hence, if CE patients were unable to exercise, their levels of depression, anxiety, and perhaps even compulsivity might increase, increasing the gap between them and non-CE patients as a result.

Self-reported CE had some influence on baseline symptomatology, but did not predict outcome at 1 year after out-patient, day and/or residential treatment. This is however far from saying that CE is an irrelevant symptom. Instead, what the current study finds, corroborated by previous longitudinal findings [6, 11], is that female patients who either started or continued with CE (approximately 25%) had considerably lower remission rates at 1-year follow-up in comparison to non-CE patients and those who ceased with CE. Due to small sample size, the picture was less clear for males. The current study was registry-based and therefore information on specific CE interventions was lacking. Also lacking was information on why patients started or continued CE, or if they informed their clinician about it. To date, there is no evidence-based treatment intervention developed to specifically tackle CE, and the way CE is approached in ED treatment seems to vary [41, 42]. As exercise is more socially accepted and perhaps seen as less detrimental than purging, we speculate that CE might risk being given less priority in treatment. Our results suggest that CE needs attention in treatment in order to improve prognosis. Cook and colleagues summarizes in a recent review 11 guiding principles in addressing CE in ED treatment [43]. They suggest individually tailored and graded exercise programs integrated into ED treatment, adjusted to the patients’ nutritional status and starting from mild intensity. They further suggest psycho-education, positive reinforcement and patient evaluation of every exercise session. Additionally, there are encouraging preliminary findings regarding a CBT-based targeted intervention, the Loughborough Exercise Activity Program (LEAP), among adult patients with AN [44]. We greatly encourage systematic research on clinical interventions for CE.

As in Levallius’ et al. adolescent sample [11], there was a subset of patients with possible denial of ED. Deniers tended to report lower overall ED symptomatology, including CE. Excluding them from analysis increased CE prevalence in both females and males, decreased the magnitude of associations between CE and ED pathology and restraint, and removed the association to negative perfectionism. Denying problematic behaviors and ED pathology seems more common in individuals who exercise excessively compared to those who do not [45]. Perhaps individuals who exercise, regardless of the compulsive aspects, primarily think of their behavior as healthy and inherently positive, and as such are less comfortable admitting potentially negative effects of their behavior. Self-reported CE may therefore underestimate both prevalence and magnitude of CE. A future direction for research in this area would be to use objective means of assessing physical activity (e.g. accelerometers), and potentially compare such data with subjective measures of CE.

### Strengths and limitations

The present study has several strengths. It is by far the largest study on CE in both female and male ED patients at all levels of care (i.e. out-patient, day and/or residential treatment), with the main ED diagnoses included. The large sample enabled simultaneous examination of associations previously examined in different studies, including prospective associations. Further, the sample was drawn from a database with nationwide coverage, high coverage ratio, and structured assessments with validated instruments, all contributing to good ecological validity and generalizability of results.

However, some limitations need to be considered. First, most of our data came from self-report measures, which requires a certain amount of introspective ability on part of the patients, as well as an ability to concentrate and to understand the questions being asked. However, where possible, we also ran the analyses using clinician-rated symptoms and found a similar pattern of results, tentatively supporting the validity of our findings. Second, CE was measured by two self-rated items from the EDE-Q, originally intended to measure presence, and frequency of driven/compulsive exercise. These items may not capture all relevant aspects of CE [5], and may, as has been mentioned, lead to underestimations of CE, especially in the AN-group where CE might be better captured by objective rather than subjective measures [23]. However, there were more possible deniers in the non-CE group than in the CE group, even if deniers were found in both groups. CE may thus be underestimated but not necessarily to a greater extent than ED symptoms in general. Also, we mainly analyzed CE dichotomously. However, the EDE-Q frequency item is strongly correlated to established CE measurements [18], and the same methodology has been used previously in adolescent EDs with similar results [11]. Also, mainly confirming findings by Levallius et al. [11], CE *frequency* provided almost no additional information on relations to pathology, characteristics, or outcome. Taken together, even if EDEQ presence of driven/compulsive exercise does not capture the nuances of CE or the objective presence/amount of physical activity, it seems adequate enough to screen for CE. Third, both self-esteem and negative perfectionism were measured by instruments originally intended to measure self-affiliation and self-blame within the SASB framework. However, item content and theoretical underpinnings have clear similarities, and most likely equivalent concepts are being measured. Fourth, information on what kind of treatments patients were given are lacking, and hence, there are numerous factors that might affect 1-year outcome other than changes in CE behavior. Relatedly, there was considerable attrition to follow-up assessment (65%). Although drop-out analyses found no differences, there might still be unknown factors affecting generalizability of results. Additionally, there was no information on what kind of exercise patients carried out, which might have provided more specific results. For instance, since some sports are more afflicted by ED psychopathology than others [46, 47], such exercise activities might be more destructive than others. Alternatively, once an ED is present and there is a compulsive component, any exercise activity might be equally destructive. Lastly, our longitudinal results may indicate that CE worsens prognosis, but could also be indicative of these groups simply maintaining more ED pathology overall over the course of treatment. For example, if we were to investigate purging in a similar manner as CE, we might find patients who fail to cease (or begin) purging displaying similar poor remission rates as our CE patients. For instance, in a study by Stiles-Shields and colleagues [48], driven exercise was clearly associated with more ED symptoms, though purging as a symptom was even more strongly associated to symptoms, and the combination of these behaviors resulted in the highest levels of symptoms.

## Conclusions

In this nationwide large-scale study, almost half of all ED patients reported CE, a behavior occurring regardless of gender, age and ED diagnosis. CE was a transdiagnostic symptom, indeed a cardinal ED symptom, which should not be ignored. The most commonly used ED assessment measures, and perhaps even the DSM diagnostic criteria, ought to be updated to better capture CE, and to alert clinicians of its relevance. In the meantime, mere presence of self-reported CE seems informative and holds clear implications. There were few differences in CE vs. non-CE patients at baseline, except that CE was associated to increased dietary restraint and overall ED-pathology. However, starting or continuing CE during treatment was associated to considerably worse remission rates. Therefore, we strongly recommend CE to be systematically assessed, addressed, and evaluated.

## Abbreviations

AN: Anorexia nervosa
AN-BP: Anorexia nervosa binge/purge subtype
ANOVA: Analysis of variance
AN-P: Anorexia nervosa purging subtype
AN-R: Anorexia nervosa restricting subtype
BED: Binge eating disorder
BMI: Body mass index
BN: Bulimia nervosa
CE: Compulsive exercise
CPRS: Comprehensive Psychopathological Rating Scale
DSM: Diagnostic and Statistical Manual of Mental Disorders
ED: Eating disorder
EDE: Eating disorder examination
EDE-Q: Eating disorder examination questionnaire
EDNOS: Eating disorder not otherwise specified
GAD: Generalized anxiety disorder
OCD: Obsessive/compulsive disorder
SASB: Structural Analysis of Social Behavior
SCID-I: Structured Clinical Interview for DSM-IV Axis I Disorders
SEDI: Structured Eating Disorder Interview
TAU: Treatment as usual

## Footnotes

1 Relations between CE and any occurrence (presence/absence) of binge and purge episodes were also examined by *χ*^2^, but neither result reached meaningful significance.

2 Nor were there any differences between CE patients and non-CE patients in presence of clinician-rated ongoing depressive episode, GAD or OCD (examined by *χ*^2^).
